# A. V. M. A. at Detroit

**Published:** 1900-07

**Authors:** 


### EDITORIALS.

### A. V. M. A. AT DETROIT.

IN this issue will be found a full outline of the programme at Detroit. Secretary Stewart has been assiduous in his work, and has secured many promised contributions on a variety of subjects that will attract the keenest attention of the profession all over the land.

The present year has been a very favorable one in veterinary circles, and the attendance should be larger than for many years. There should be many new faces at Detroit from a section of the United States and Canada that has not been well enough represented in Association circles in past years. While the Northwest may regret that she was not favored with the meeting, still there will be few of her devoted Association members absent at Detroit. From Ohio and Indiana there should be an increased interest and attendance, and there will be on the programme more than ample to repay the remotest veterinarian in our land for every sacrifice made in attending the 1900 convention. Let there be but few stay-at-homes. Let there be many applicants knocking for admission.

